# The effect of cavity disinfectants on the sealing ability of dentin bonding system: An *in vitro* study

**DOI:** 10.4103/0972-0707.57634

**Published:** 2009

**Authors:** Vivek Sharma, Mohan T Nainan, Vasundhara Shivanna

**Affiliations:** Departments of Conservative Dentistry and Endodontics, College of Dental Sciences, Davangere, Karnataka, India

**Keywords:** Benzalkonium chloride, cavity disinfectant, chlorhexidine gluconate, dentin-bonding resin, iodine-potassium iodide/copper-sulphate, microleakage

## Abstract

**Aim:**

This study was conducted to determine the effect of three cavity disinfectants (chlorhexidine gluconate based-Consepsis; benzalkonium chloride-based Tubulicid Red, iodine-potassium iodide/copper-sulphate based Ora-5) on the microleakage of a dentin bonding system, Clearfil SE Bond.

**Materials and Methods:**

Class V cavities were prepared on 45 extracted molars. The respective experimentalgroups were treated with cavity disinfectants and Clearfil SE Bond. Preparations without cavity disinfectants served as negative control and those with neither disinfectant nor dentin bonding resin application served as positive controls. After the cavity preparations were restored with resin composite (Clearfil APX), the specimens were subjected to dye penetration. Statistical analysis was performed using ANOVA (Kruskal-Wallis) test.

**Results:**

Unlike Conspesis and Tubulicid Red, Ora-5 exhibited significantly higher microleakage and adversely affected the sealing ability of Clearfil SE bond. Only Consepsis and Tubulicid Red could be used as cavity disinfectants with Clearfil SE bond, without its sealing abilities being adversely affected.

**Conclusions:**

1) Consepsis and Tubulicid Red can be used as cavity disinfectants with Clearfil SE Bond, without the sealing ability of Clearfil SE bond being affected. 2) Ora-5 is not an appropriate disinfectant to be used with this dentin bonding system, because it alters its sealing ability.

## INTRODUCTION

The setting reaction of resin composites involves polymerization shrinkage that may lead to the formation of a contraction gap at the tooth restoration interface. This gap can result in the passage of bacteria, fluids, or ions between the cavity wall and the resin composite, a process which is known as microleakage. Although many new bonding systems for reducing the size and incidence of gap formation following placement of resin composite restoration have been introduced, microleakage, especially at the dentin (cementum) aspect of restoration, remains a problem of clinical significance.[1–3] Microleakage has been demonstrated as a factor in hypersensitivity, secondary caries and pulpal pathology.[4]

Restorative procedures such as cavity preparation are used to remove the infected dentin and make space for the restorative materials. The successes of these procedures depend on the effective removal of infected dentin, prior to the placement of the restorative material. The problem associated with microleakage can be magnified by incomplete sterilization of the preparation, as a consequence of failure to mechanically remove the infected tooth structure.[5] Histological and bacteriologic studies have shown that only a small proportion of the teeth are sterile after cavity preparation and that bacteria left in the cavity preparation could survive for longer than a year.

Therefore, after removal of the carious dentin, it is important to eliminate any remaining bacteria that may be present on the cavity walls, in the smear layer, at the enamel-dentin junction, or in the dentinal tubules.[6]

Today, the application of disinfectants after cavity preparation and before tooth restoration is gaining acceptance. It eliminates potential risks due to bacterial activity.[7,8] However, there is concern about the use of cavity disinfectants with dentin bonding agents, since they may alter the ability of the hydrophilic resin to seal the dentin.[9] Contrary to this concern, it has been suggested that cavity disinfectants can improve the sealing ability of dentin-bonding agents by remoistening the cavity, prior to placing a dentin-bonding agent that bonds to damp tooth structure.

The purpose of this study was to evaluate the effect of three different cavity disinfectants on the microleakage of a non rinsing dentin-bonding system, Clearfil SE Bond (Kuraray).

## MATERIALS AND METHODS

Forty five extracted human molars, free of cracks, caries and restorations on visual inspection, were used for the study. The teeth were scraped of any residual tissue tags, kept in 2.6% sodium hypochlorite solution and rinsed under running water for 15 minutes each. Later, they were cleaned with pumice and stored in normal saline at 40°C until use.

Class V cavity preparations were prepared on the facial and lingual surfaces of each tooth, with a cylindrical diamond bur, in a high speed handpiece utilizing water-spray coolant. Standardized preparations were obtained by making cavity preparations that were approximately 2 mm wide, 1.5 mm deep and 4 mm long, paralleling the cementoenamel junction (CEJ). The gingival half of the preparation was extended 1 mm below the CEJ. No bevels were used in the preparation. Cavosurface walls were then finished and polished.

Each preparation was rinsed with distilled water for 20 seconds and dried with compressed air for 20 seconds.

The teeth were then randomly divided into five groups [Table 1] as follows:

Group I consisted of ten teeth (20 cavity preparations) treated with chlorhexidine based cavity disinfectant solution (Consepsis, Ultradent USA), followed by the application of a dentin bonding system (Clearfil SE Bond).

Group II consisted of ten teeth (20 cavity preparations) treated with benzalkonium chloride based cavity disinfectant solution (Tubulicid Red, Dental Therapeutics AB, Sweden), followed by the application of a dentin bonding system (Clearfil SE Bond).

**Table 1:** Experimental groups used in the study

Groups	Disinfectant treatment	Dentin bonding system
I	Consepsis	Clearfil SE bond
II	Tubulicid red	Clearfil SE bond
III	Ora-5	Clearfil SE bond
IV*	None	Clearfil SE bond
V†	None	None

* = Negative control and

* † = Positive control

Group III consisted of ten teeth (20 cavity preparations) treated with iodine-potassium iodide/copper sulphate based cavity disinfectant solution (Ora-5, McHenry Lab, Texas, USA), followed by the application of a dentin bonding system (Clearfil SE Bond).

Group IV (negative control) consisted of ten teeth (20 cavity preparations) used without any cavity disinfecting solution treatment; however, a dentin bonding system (Clearfil SE Bond) was applied.

Group V consisted of five teeth (10 cavity preparations), used without either a cavity disinfecting solution treatment or a dentin bonding system.

The preparations without cavity disinfectant application were used as the negative controls and the cavities in which neither disinfectant nor dentin bonding system was applied, served as positive controls.

In the respective test groups, cavity disinfectants were applied with a sterile brush applicator for 20 seconds; any excess disinfectant was removed by five seconds of light air drying, to prevent desiccation [Table 2].

After cavity disinfection, the dentin bonding system (Clearfil SE Bond) was applied to the appropriate groups, in keeping with the manufacturer's instructions. The cavity preparations were exposed to one drop of primer application for 20 seconds and gently air dried. A layer of bonding resin was applied to the preparation with a brush, spread gently with air and cured for 10 seconds. The cavity preparations were restored with a resin composite (Clearfil APX) by light curing for 60 seconds. The cavosurface margins were then finished with a finishing bur and 3M USA discs.

All the teeth were stored in distilled water for 24 hours, at 37°C, and subjected to 1,000 thermal cycles between water baths of 50°C and 550°C, with a dwell time of 30 seconds. The teeth were then subjected to dye leakage tests.

**Table 2:** Experimental groups used in the study

Product	Active ingredient	Manufacturer
Consepsis	2% chlorhexidine gluconate	Ultradent, USA
Tubulicid red	0.1% benzalkonium chloride, 0.2% EDTA, 1% sodium fluoride	Dental therapeutics, AB, Sweden
Ora-5	0.3% iodine, 0.15% potassium iodide, 5.5% copper sulphate	McHenry labs, Texas, USA

All the teeth to be subjected to dye-leakage tests were covered with two coats of nail varnish to within 1 mm of the tooth-restoration margin, after the root apices were sealed with modelling wax. The specimens were immersed in India ink, in separate sealable glass vials, at 37°C for 24 hours.

After staining, the teeth were rinsed, in order to remove any residual stain, and the radicular parts of the teeth were cut 4.5 mm below the CEJ. Coronal parts were sectioned mesiodistally and then buccolingually, in the approximate center of the restoration, with a low speed diamond saw.

Microleakage was assessed for both occlusal (enamel) and gingival (cementum) margins, using a stereomicroscope at original magnification ×16.

The depth of the stain (dye leakage) was judged according to the following scale:

0 – No leakage

1 – Penetration less than one half of the length of occlusal/gingival wall

2 – Penetration greater than one half of the length of occlusal/gingival wall

3 – Penetration up to and along the axial wall

4 – Penetration within the pulp

A nonparametric analysis of variance (ANOVA) test (Kruskal-wallis) was used to determine statistically significant differences among the groups. Pairwise comparisons between groups was made using the Mann Whitney Wilcoxon U Test for nonparametric data.

Occlusal and gingival margins within the treatment groups were compared using Wilcoxon matched pairs signed rank test. The level of significance was established as *P* < 0.05, for all the tests.

**Figure 1:**
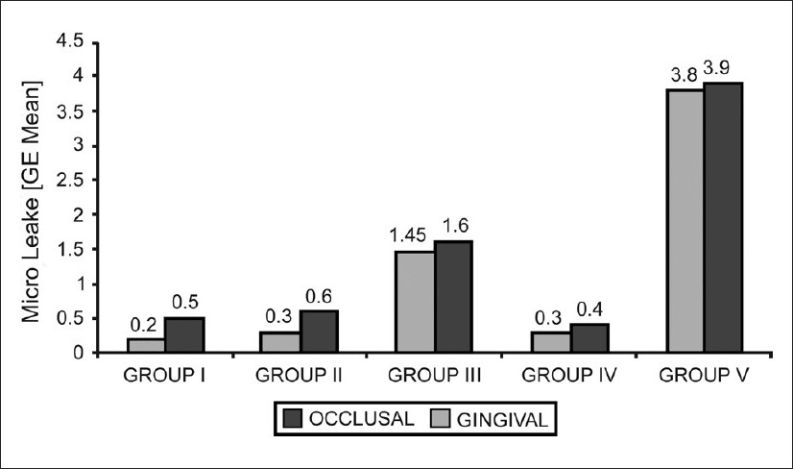
Mean microleakage scores seen in all the groups in the occlusal and gingival walls respectively

## RESULTS

### Interpretation of data

Almost all the positive control specimens showed maximal leakage (score 4), whereas the experimental groups and negative controls exhibited less leakage [Figure 1].

The Kruskal-Wallis test revealed that the differences were highly significant at each (occlusal and gingival) margin.

When all possible pairwise comparisons were made in the occlusal wall, there were no statistically significant differences among groups 1, 2 and 4 (negative control), whereas all the other combinations showed highly significant differences, suggesting that the sealing ability was not adversely affected with Tubulicid Red and Consepsis cavity disinfectants.

Similarly, all possible pairwise comparisons made in the gingival wall showed that there were no statistically significant differences among groups 1, 2 and 4 (negative control), whereas statistically significant differences were found between all the experimental groups and both the negative and positive controls. However, when the experimental groups were compared among themselves, there were very significant statistical differences, except between group 1 and group 2, which suggested that there were marked differences in the sealing ability of Tubulicid Red and Consepsis cavity disinfectants on the one hand and Ora-5 cavity disinfectants on the other.

Although the occlusal margins showed much less leakage than the gingival margins, there was no statistically significant differences between the occlusal and gingival scores of the groups (*P* > 0.05).

## DISCUSSION

Historically, it was suggested that dentin should be sterilized before the placement of any restorative material. Many chemicals, such as silver nitrate precipitated with eugenol, thymol, and potassium ferrocyanide, had been proposed for this purpose.[10] The rationale prevailing for this was that any residual microorganisms should be eliminated in order to prevent the potential propagation of caries. Today, it is known that these chemicals are irritating to the pulp when applied to the dentin surface.[10] Thus, any chemical that is capable of destroying microorganisms may also have a detrimental influence on the pulp.

The use of Tubulicid, benzalkonium chloride-based disinfectant has been proposed for disinfecting the cavity preparation, prior to its restoration. A study had reported that neither were bacteria seen on the cavity walls nor were pulpal reactions observed when the cavities had been treated with tubulicid before their restorations.[11]

The Tubulicid Red used in our study contains ethylene diamine tetracitic acid (EDTA) for the removal of smear layer and benzalkonium chloride, which has an antibacterial effect. It also contains a high concentration of sodium fluoride and has been claimed to accomplish three goals in one treatment – cleaning, disinfection, and impregnation with 20% reduction in dentinal permeability.[12]

Most of the current generation disinfectants contain 2% chlorhexidine gluconate as the primary active ingredient, which is an antiseptic with a wide spectrum of action. Consepsis used in our study contains 2% chlorhexidine gluconate and has been reported to have better antimicrobial activity.

Ora-5 used in our study is commercially available iodine – potassium iodide/copper sulphate (I2-KI/CuSO_4_) based oral disinfectant. Several studies in humans have shown that I2-KI solutions can reduce the streptococcus mutans levels on smooth surface for prolonged periods.[11]

The use of cavity disinfectants after tooth preparation and before the application of dentin-bonding agents could help reduce the potential for residual caries and postoperative sensitivity.[13] However, any positive benefits would be negated if the solutions significantly increased the amount of microleakage, by interfering with the bonding agent's interaction with dentin.

The results of our study indicate that treatment of cavity preparations with Tubulicid Red, before the application of Clearfil SE bond, did not adversely affect the sealing ability of the dentin bonding system (Clearfil SE bond). Our results are in accordance with a study conducted by Derhami *et al.*[14]

In our study, Consepsis solution also did not adversely affect the sealing ability of Clearfil SE bond. This finding is in accordance with a study conducted by Meiers *et al*.[8] However, the results of our study are conflicting with another study which investigated the effect of chlorhexidine gluconate on the sealing ability of dentin bonding systems to prevent microleakage in primary teeth.[9]

These differences can be attributed to the varying thickness of the hybrid layer, which develops in primary and permanent teeth. Hybrid layer produced in primary teeth is significantly thicker than in permanent teeth, suggesting that primary tooth dentin is more reactive to acid conditioning. The increased thickness of the hybrid layer in primary teeth (25 to 30%) and the subsequent lack of complete penetration of adhesive resin into previously demineralized dentin may contribute to the lower bond strengths to primary dentin reported in the literature.

The presence of statistical difference in our study, between the negative control and the experimental group III, which was treated with Ora-5 before the application of the dentin adhesive systems, in both occlusal and gingival marginal leakage scores, showed that Ora-5 adversely affected the sealing abilities of Clearfil SE bond. The results of our study was in accordance with another study conducted by Meiers *et al*.[8]

Therefore, it can be concluded from our study that the use of cavity disinfectants with resin composite restorations appears to be material specific, with regard to interaction with the ability of various dentin bonding systems to seal dentin.

## CONCLUSION

The following conclusions can be drawn from the results of this study:

1. Consepsis and Tubulicid Red can be used as cavity disinfectants with Clearfil SE Bond, without the sealing ability of Clearfil SE bond being affected.
2. Ora-5 is not an appropriate disinfectant to be used with this dentin bonding system, because it alters its sealing ability.

## ACKNOWLEDGEMENT

The authors are thankful to Mr. Kenneth Larsson of Dental Therapeutics AB, Sweden, Mr. Mac Lee of McHenry Lab, Texas, USA, Mr. Nathan D Johnson of Ultradent USA, and Tracom Services Pvt. Ltd. India, for providing cavity disinfectants and other materials related to the study.
